# Unleashing the potential of a low CpG *Passer* transposon for superior CAR-T cell therapy

**DOI:** 10.3389/fimmu.2025.1541653

**Published:** 2025-02-06

**Authors:** Jianyao Zeng, Yan Sun, Yuan Fang, Xiaodie Wang, Qian Huang, Pingjing Zhang, Meiqi Shao, Pei Wang, Jingbo Cheng, Meng Di, Tao Liu, Qijun Qian

**Affiliations:** ^1^ School of Medicine, Shanghai University, Shanghai, China; ^2^ Innovative Drugs Business Group, Shanghai Cell Therapy Group, Shanghai, China; ^3^ Shanghai Mengchao Cancer Hospital, Shanghai University, Shanghai, China

**Keywords:** methylation, CpG motif, chimeric antigen receptor T cell, transposon, inflammation

## Abstract

**Background:**

To date, the non-viral vector Chimeric Antigen Receptor (CAR) T cell preparation platform, exemplified by transposons, has demonstrated significant potential in tumor immunotherapy and yielded positive results in multiple clinical trials. Nonetheless, non-methylated CpG sequences within plasmid DNA can elicit an inflammatory response via Toll-like receptor 9 (TLR9) during CAR-T cell preparation, adversely affecting transgene expression. Additionally, de novo DNA methylation programs promote T cell exhaustion, which poses a significant limitation for CAR-T cell therapy applications.

**Methods:**

High-throughput liquid protein chip and CBA analyses were utilized to determine the expression levels of inflammatory factors. Flow cytometry and luciferase reporter assays were employed for mutation screening. BALB/c mice and M-NSG mice were used to evaluate the inflammatory response and efficacy of LCG CAR-T *in vivo*, with TIL grouping detected via immunohistochemistry.

**Results:**

In this study, we modified the newly discovered Passer (JL) transposon to construct a low-CpG content transposon for CAR-T cell (LCG CAR-T cell) preparation. *In vitro* experiments demonstrated that LCG CAR-T cells prepared using this new transposon exhibited stronger cytotoxicity. In animal models, LCG CAR-T cells significantly inhibited tumor growth and increased the populations of CD4+CAR-T cells and tumor-infiltrating lymphocytes. Furthermore, LCG CAR-T cells modulated pro-inflammatory cytokine release, thereby reducing *in vivo* inflammatory responses and surpassing the effects observed with unmodified CAR-T cells.

**Conclusions:**

Collectively, our results demonstrate the high safety and efficacy of non-viral, low CpG Passer transposon CAR-T cells, offering new avenues for improving CAR-T cell efficacy while minimizing *in vivo* inflammation.

## Graphical Abstract

In this study, the authors identified a Low-CpG *Passer (JL)* transposon as an effective platform for LCG CAR-T cells. LCG CAR-T cells reduced inflammatory cytokines, enhanced T cell infiltration, increased CD4+ CAR-T cells, and elevated IFN-γ secretion, leading to a pronounced antitumor effect. The results indicate that the low-CpG *JL* transposon system holds significant clinical application value.

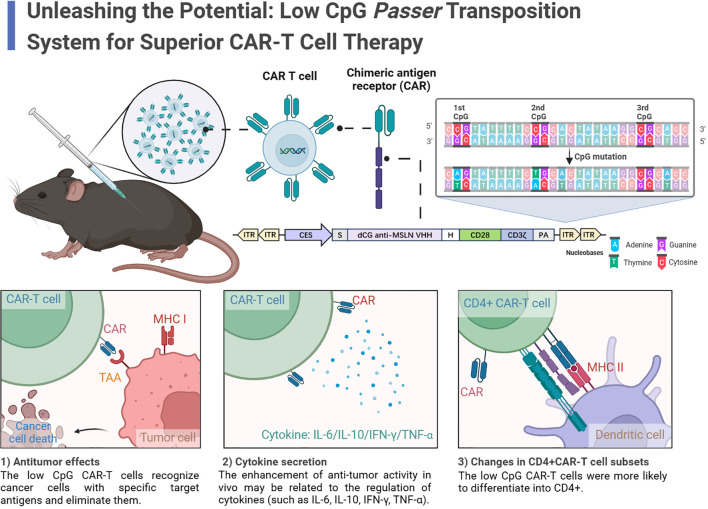

## Introduction

Chimeric Antigen Receptor (CAR) T cell therapy has transformed the landscape of malignant tumor immunotherapy, fundamentally altering traditional cancer treatment strategies. However, the reliance on viral vectors for T-cell transfection poses limitations, hindering the broader application of this promising therapeutic approach. The use of non-viral vectors for CAR-T cell preparation is emerging as a more versatile and sustainable alternative in next-generation therapies.

Transposable elements (TEs), first discovered in maize by Barbara McClintock in the 1940s (1), are mobile DNA sequences consisting of gene fragments flanked by inverted terminal repeats (ITRs) and transposase. This enzyme facilitates the excision of the transposon from its original DNA site and its integration into a new genomic location. TEs can be divided into retrotransposons and cut-and-paste two major categories transposition mechanisms (2). Cut-and-paste transposons necessitate transposase recognition of two ITRs to excise the DNA transposon from its source and integrate it elsewhere (3). This inherent ability to insert DNA makes cut-and-paste transposons powerful tools for genome manipulation (4–7).

Virus-derived DNA specific reactions frequently hinder CAR expression (8–10). Additionally, viral production for clinical applications typically takes 2-3 weeks and is often associated with high costs, influencing the final price of CAR-T products (11). The complexity and cost associated with preparing CAR-T cells using transposon vector systems are significantly lower than those linked to viral vectors, with costs being 5-10 times less (12). Transposon systems can express transgenes in both activated and resting T cells (13) and increasing evidence underscores the feasibility and safety of CAR-T cell preparation platforms based on transposon systems (11). For instance, Monjezi et al. successfully utilized enhanced *Sleeping Beauty* (*SB*) transposons to prepare CAR-T cells, resulting in robust anti-CD19 CAR-T cells responses (14). The *SB* vector has demonstrated long-term *in vivo* expression in multiple preclinical studies, supporting the utility of transposon systems in CAR-T cell construction (15).

**Figure 1:**
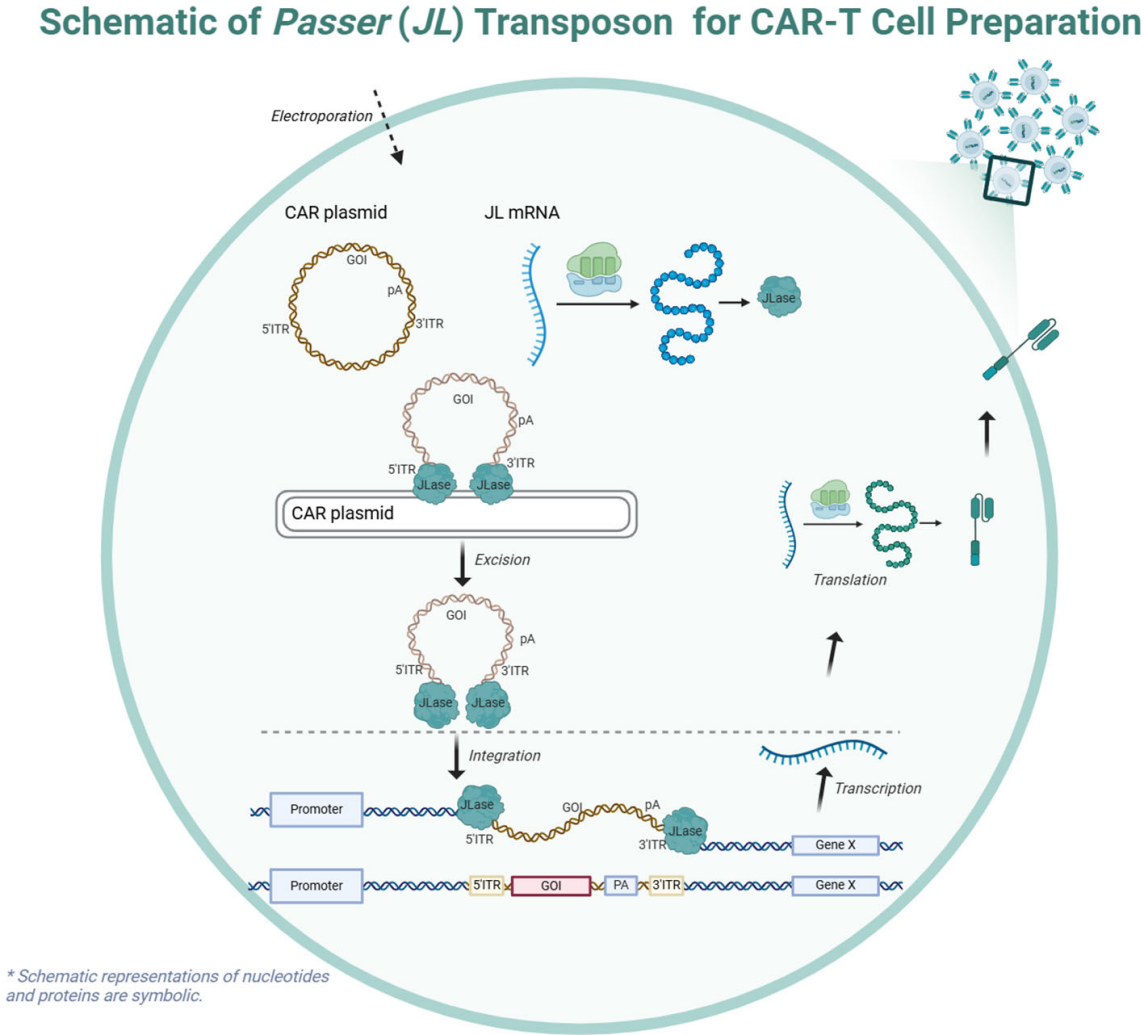
Schematic of the *Passer (JL)* Transposition System.

In this study, we focus on the newly discovered *Passer* (*JL*) transposon, which belongs to the *pogo* superfamily and is predominantly found in animal genomes. The *JL* transposons are characterized by a cut-and-paste mechanism and feature transposase with highly conserved catalytic motifs (DD35D) and relatively short ITRs (16), compared to other highly active transposons such as *ZB* (17) and *SB* (18). The schematic diagram of CAR-T cell preparation using the *JL* transposon system is shown in the **Figure 1**.

Despite the demonstrated efficacy of CAR-T cell therapies utilizing non-viral vectors, T cell exhaustion remains a critical challenge. DNA methylation primarily involving the addition of a methyl group to the fifth position of cytosine within CpG dinucleotides, resulting in the formation of 5-methylcytosine (5mC) and establishing a symmetrical methylation pattern across the DNA double helix (19). For example Brooke Prinzing et al. found that DNMT3A deficiency could prevent *de novo* methylation in human CAR-T cells, allowing them to maintain their differentiation ability and proliferate effectively in the presence of antigens (20). This underscores the critical role of methylation levels in sustaining CAR-T cell survival and function (21).

During CAR-T cell preparation, foreign nucleic acids may be recognized by the Toll-like receptor (TLR) family, triggering a cascade of innate immune responses (22). TLR9 is specifically implicated in recognizing unmethylated CpG dinucleotide motifs (23) and TLR9-related mRNA and protein are expressed in various immune cells (23–28). These regulators regulate downstream signaling pathways by recruiting the MyD88 adaptor protein, which stimulates the production of inflammatory factors (22, 29, 30). Stephen C. Hyde et al. demonstrated that using CpG-free pDNA expression vectors could achieve sustained transgene expression with minimal inflammation, resulting in improved therapeutic efficacy (31). Consequently, we modified the *JL* transposon to eliminate CpG sequences within the ITRs, thereby reducing the adverse effects of inflammation and methylation on CAR-T cell function.

In this study, we present a novel low-CpG *JL* transposon-based CAR-T cell preparation platform. Our results indicate that CAR-T cells generated using the modified *JL* transposon exhibit enhanced antitumor activity both *in vitro* and *in vivo* compared to the unmodified system. The low-CpG *JL* transposon system holds potential for the preparation of various types of CAR-T cells, offering significant clinical application value.

## Materials and methods

### Cell lines

Jurkat and SK-OV-3 cells were obtained from the American Type Culture Collection, while CHO-K1 cells were sourced from the Cell Bank of the Chinese Academy of Sciences. All cell lines were authenticated by short-tandem-repeat profiling. SK-OV-3 cells were maintained in DMEM (corning) supplemented with 10% FBS (Gibco). Jurkat cells were cultured in RPMI-1640 (Hyclone) with 10% FBS (Gibco), and CHO-K1 cells were kept in F12K (Gibco) supplemented with 10% FBS (Gibco). An SK-OV-3 cell line stably expressing Fluc was established through electroporation, followed by selection with puromycin. Mycoplasma contamination was regularly assessed to ensure cell lines integrity.

### Plasmid construction and amplification

The plasmid employed in this study was engineered from the pTini vector developed in our laboratory. ITR sequences from the *JL* transposon were introduced at both termini of the target gene. Upon co-transfection, the target gene is integrated into the host cells, mediated by the *JL* transposase transcribed from *JL* mRNA. The ITR was mutated *via* rolling circle amplification, with primers and target gene sequences synthesized by GenScript. The MSLN CAR comprises the VHH domain targeting the MSLN antigen, a hinge region, the CD28 domain, and the CD3ζ domain (CN116410332A). Plasmid amplification was achieved by transforming modified GT-115 competent cells, followed by medium-scale plasmid extraction using the NucleoBond Xtra Midi EF kit (Macherey-Nagel). The final construct was validated through DNA sequencing performed by GenScript.

### Isolation and expansion of human primary T cells

Fresh peripheral blood mononuclear cells (PBMCs) from healthy donors were provided by Shanghai AoNeng Biological Technology Co., Ltd. Recruitments of healthy human blood donors were approved by Shanghai Zhaxin Traditional Chinese Ethics Committee and Western Medicine Hospital, with all donors providing informed consent. hPBMCs were collected via apheresis and isolated using density gradient centrifugation with Ficoll (Sigma-Aldrich). T cells were activated using T Cell TransActTM (Miltenyi Biotec) at a 1:100 dilution in AIM-V medium (Gibco) supplemented with 2% human AB serum or CTS Immune Cell Serum Replacement (Thermo Fisher) and recombinant human IL-2 (100 U mL^-1^). Cells were harvested once the required cell count for administration was achieved, followed by washing and formulation.

### Human primary T cell electroporation

Electroporation was performed 2-3 days after T cell stimulation, following the Lonza 4D manufacturer’s protocol. In brief, 1×10^7^ prewashed T cells were resuspended in 100 μL electroporation buffer P3. A total of plasmid DNA (4 µg) and *JL* transposase mRNA (5 µg) were added to the cell suspension before electroporation. Cells were then transferred into electroporation cuvettes. Programme EO115 was chosen for electroporation. After electroporation, cells were immediately supplemented with a prewarmed medium and transferred from the cuvettes.

### Transposition assay in CHO-K1 cells by GFP or Fluc

CHO-K1 cells were nucleofected with 2b-Nucleofector Reagent (Lonza # VVPA-1002) following the manufacturer’s protocol. Specifically, 1.5×10^6^ CHO-K1 cells were cotransfected cells with plasmid DNA (4μg) containing the GFP (or Fluc) expression cassette and *JL* transposase mRNA (5μg). Following transfection, the cells were cultured over two weeks to assess the stability of GFP (or Fluc) expression in CHO-K1 cells. Subsequently, the cells were analyzed using fluorescence microscopy (OLYMPUS IX73) and flow cytometry (Cytek Northern Lights # NL1213), with passaging occurring every three days.

### Jurkat cells electroporation

Jurkat cells were electroporated using a Lonza 4D electroporation system following the manufacturer’s instructions. In brief, 3×10^6^ Jurkat cells were resuspended in 100 μL electroporation buffer (Lonza SE Cell Line 4D), and 4 µg of plasmid DNA and 5 μg of *JL* transposase mRNA were added to the cell suspension. Electroporation was performed using program SE-CL120. Post-electroporation, cells were immediately supplemented with prewarmed medium and transferred from the cuvettes.

### Luciferase reporter assay

At day 3/6/9/12 after electroporation, the hPBMC (or CHO-K1) cells were collected for luciferase activity measurements using the Fluc reporter assay system following the manufacturer’s protocol. The cells were resuspended and counted in Dulbecco’s Phosphate-Buffered Saline (DPBS). We seeded the cells at a density of 4 × 10^4^ cells per well in a volume of 50 μL in a white-bottom 96-well plate. Using a multichannel pipette, add D-luciferin potassium salt to the 96-well plate, avoiding light exposure, with a volume of 50 μL per well. Incubate the plate at room temperature in the dark for 5 minutes to allow for substrate equilibration. Proceed with the detection using a microplate reader. Fluc expression was measured at 450 nm using an EnVision multilabel reader (PerkinElmer, Seer Green, UK). The normalized intensity of Fluc was calculated as the ratio of Fluc expression in the treatment group to that in the Positive Control group.

Cytotoxicity was detected by luciferase reporter assay. CAR-T cells were co-cultured with SK-OV-3-Luc cells at the indicated effector-to-target cell ratios (E:T)=1:1/1:2/1:4/1:8/1:16. The killing efficiency of CAR-T cells on tumor cells was calculated by the following formula: Killing rate of tumor cells = 1- Treatment Group/Tumor.

### Flow cytometry

CAR and membrane protein expression were determined by flow cytometry. Cells were prewashed and incubated with antibodies for 30 min at 4 °C. After washing twice, samples were run on NL-CLC 1L Flow Cytometer (CYTEK) and CytoFLEX Flow Cytometer (Beckman Coulter) and analyzed with FlowJo software. The following antibodies were used: CD3e Monoclonal Antibody, CD8a Monoclonal Antibody, PerCP-Cy5.5, Alexa Fluor488 anti-human CD4 Antibody, Streptavidin-PE, BD™ CD62L PE (all from BD Biosciences), APC anti-human CD3 Antibody, Brilliant Violet 421™ anti-human CD45RA antibody (all from BioLegend) and PE-MonoRab™ anti-VHH nanobody (GenScript). For CAR expression detection, biotinylated human MSLN(GenScript) and PE streptavidin (BioLegend) were added sequentially, or PE-MonoRab™ anti-VHH nanobody (GenScript) was used. For clinical samples, peripheral blood cells were stained with antibodies, followed by the addition of Lysis Buffer (BD Biosciences) before being run. The percentage of CAR+ cells was analyzed in CD45+CD3+ gated cells.

### Cytokine release assay

For *in vivo* cytokines evaluation of the unmethylated CpG sequences, sera obtained from mice were analyzed for IFN-γ, IL-1β, IL-6 IL-10, IL-12p70 and TNF-α using the ProcartaPlex-06, High-throughput liquid protein chip (Thermo Fisher) following the manufacturer’s instructions. Data were collected using the Luminex 200 (Thermo Fisher). Cytokine concentrations were calculated using a five-parameter nonlinear regression equation to fit the standard curve.

For *in vivo* evaluation of CAR-T infusion products, sera obtained from mice were assessed for IL-6, IL-10, IFN-γ, and TNF-α using the BD cytokine cytometric bead array (CBA) human Th1/Th2 cytokine kit II (BD Biosciences, USA) following the manufacturer’s protocol. Data were acquired using a Navios flow cytometer (Beckman Coulter, USA) and analyzed using FCAP Array v3 software Analyze software. Conditioned media or sera were stored at -80 °C in 1.5 mL tubes until use.

### *In vivo* experiments

For the study investigating the correlation between CpG content and the expression levels of inflammatory cytokines, 6-8 week-old female BALB/c mice (Phenotek Biotechnology, Shanghai) were administered either CpG-free plasmid (0 CpG) or non-CpG-free plasmid (304 CpGs) via intravenous (i.v.) or intraperitoneal (i.p.) injection. These plasmids expressed the Fluc reporter gene. Polyethylenimine (PEI) and 5% glucose served as control treatments. The grouping day was designated as Day 1. PEI-plasmid was administered to different groups starting from Day 0. The concentration of PEI-plasmid was 100 μg mL^-1^ and the volume was 200 μL. Blood samples were collected from all mice 6 hours post-administration and centrifuged to isolate serum for subsequent cytokine detection. The weight of the experimental animals was recorded on the grouping day and daily thereafter. Bioluminescence imaging was performed using the IVIS Imaging System and software (PerkinElmer). Daily observations included mortality and any drug-related effects on the mice, such as changes in activity, food and water intake, weight loss, coat appearance, and other clinical symptoms. Mice were euthanized according to experimental protocols or when they reached predefined endpoints as established by the Institutional Animal Care and Use Committee.

To evaluate the anti-tumor efficacy of LCG CAR-T cells, 6-8 week-old M-NSG female mice (Shanghai Model Organisms) were injected i.v. with 1 × 10^7^ Fluc-transduced SK-OV-3 cells. At 4 days after tumor engraftment (the mean tumor volume reached 89.65 mm^3^), mice were divided into four groups and inoculated with LCG CAR-T cells, normal CAR-T cells, mock T cells and PBS. Mice received intravenous injection of 1 × 10^6^ CAR-T cells (first dose) and 5 × 10^6^ CAR-T cells (second dose). Bioluminescence imaging was again utilized for quantitative analysis of tumor growth. The experiments were conducted at the Shanghai Research Center of Southern Model Organisms.

All animal experiments were conducted in accordance with the Guide for the Care and Use of Laboratory Animals and were approved by the IACUC of Shanghai University Center for Animal Research and Phenotek Biotechnology (Shanghai) Co., Ltd.

### Immunohistochemistry

Immunohistochemistry (IHC) analysis was undertaken on formalin-fixed, paraffin-embedded tissue sections. The deparaffinization process utilized xylene, followed by rehydration through a graded alcohol series. Endogenous peroxidase activity was inhibited using 3% hydrogen peroxide. Antigen retrieval was performed using EDTA buffer (pH 9.0). After rinsing with PBS, tissue sections were incubated with antibodies against human CD3 (Biolynx) for IHC staining. Staining was carried out on an automated immunostainer (Leica Bond-III, Dako Autostainer Link 48) using a Bond Polymer Refine Detection system.

The IHC Profiler plugin in Fiji was utilized for the quantitative analysis of immunohistochemical staining. Initially, the images were converted to 8-bit grayscale to ensure consistency in the analysis. The plugin categorized staining intensity into four distinct levels: high positive, positive, low positive, and negative. Appropriate thresholds were established to differentiate stained areas from background. The plugin calculated the area and percentage for each intensity level, yielding a comprehensive quantitative assessment, which was subsequently analyzed to determine overall staining intensity.

### Statistics

Data were analyzed by unpaired t-test for the two-group comparison. One- or two-way analysis of variance (ANOVA) or Kruskal-Wallis test and Dunnett’s or Games-Howell multiple comparisons test were used for multiple groups comparison. The results were presented as the mean ± S.D. or the mean ± S.E.M. as described in the figure legends. Statistical analyses were performed using GraphPad Prism 9.0 and considered significant at *p < 0.05, **p < 0.01, ***p < 0.001, ****p < 0.0001 and NS inficates not significant.

## Results

### CpG-free plasmids enable sustained expression of the target gene *in vivo*

We prepared jetPEI/DNA complexes with varying N/P ratios to optimize *in vivo* transfection. The plasmid structure is illustrated in **Supplementary Figure S1A**. An N/P ratio of 6 was identified as optimal for both experimental and control groups, demonstrating the highest transfection efficiency; thus, N/P = 6 was selected for subsequent mouse experiments (**Supplementary Figure S1B**).

**Figure 2:**
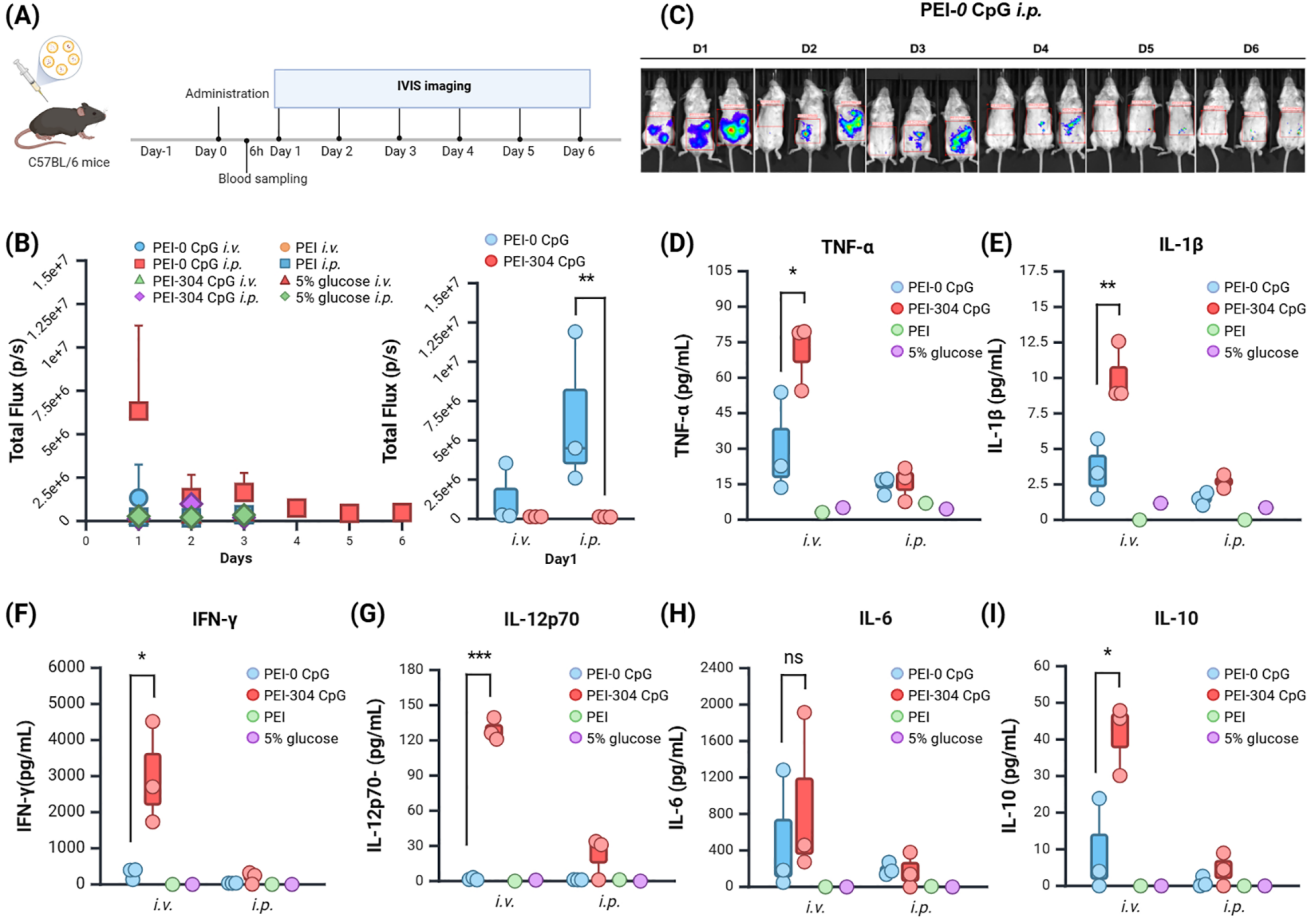
Expression and Impact of Plasmids with Different CpG Content in Mice. **(A)** Schematic of the administration protocol. **(B)** Calculation of tumor single or total Flux (unit: p/s) using *in vivo* imaging software and **(C)** Imaging. **(D–I)** Levels of **(D)** TNF-α, **(E)** IL-1β, **(F)** IFN-γ, **(G)** IL-12p70, **(H)** IL-6, and **(I)** IL-10 in serum of mice from different groups. n = 3, Mean ± SEM; *p value < 0.05, **p value < 0.01, ***p value < 0.001, ns represents no significance.

To assess the sustained expression of CpG-free plasmids *in vivo*, the BALB/c mice were randomly assigned to groups (**Figure 2A**). Following a single injection, *in vivo* fluorescence imaging was conducted on days 1 through 6 post-administration. Different plasmids and administration routes did not significantly affect the body weight of the mice (**Supplementary Figure S1C**), although the body weight in the PEI-304 CpG i.v. group showed a slight decrease, accompanied by abnormal fur erection observed on day 3.

The total fluorescence intensity (Total Flux) indicated that the PEI-CpG-free groups expressed fluorescence on day 1, while the other groups exhibited weak fluorescence from days 1 to 3 (**Figure 2B**). Compared to other groups, the PEI-CpG-free i.p. group exhibited stronger and more prolonged fluorescence expression, indicating that CpG-free plasmids facilitate longer-term expression of the target gene relative to CpG-containing plasmids (**Figure 2C**).

### CpG-free plasmids mediate a low-inflammatory cytokine environment *in vivo*

The peripheral blood of mice was collected at 6 hours after administration, and cytokine levels (IFN-γ, IL-1β, IL-6, IL-10, IL-12p70, TNFα) in the serum were quantified using a high-throughput liquid protein chip. The PEI-304 CpG i.v. group exhibited higher levels of all cytokines compared to both the CpG-free group and the control group, with significant differences in IFN-γ, IL-1β, IL-10, IL-12p70, and TNFα (**Figures 2D–I**). This finding correlates with the observed abnormal fur presentation in PEI-304 CpG i.v. group mice on day 3. In contrast, the CpG-free plasmid group, particularly the i.p. injection cohort, demonstrated reduced levels of all inflammatory cytokines. These results indicate that CpG-free plasmids are associated with lower inflammatory cytokine levels in mouse serum, thereby supporting long-term gene expression in a low-inflammatory environment.

### Mutation screening reduced the CpG dinucleotide content in the ITR region of the *JL* transposon

**Figure 3:**
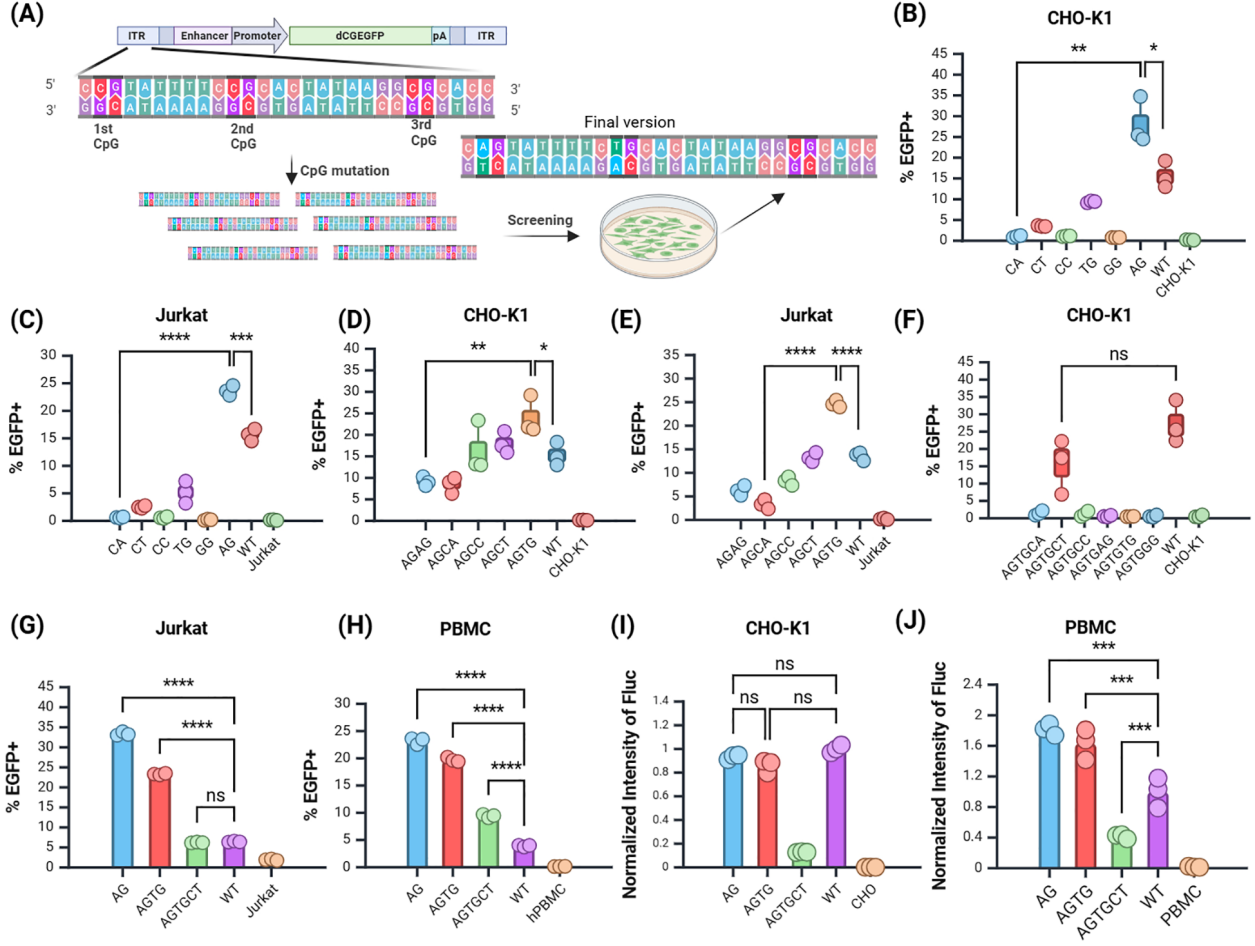
Screening of the JL Transposon ITR Mutants. **(A)** Schematic of mutation screening. **(B, C)** Screening of the first site in **(B)** CHO-K1 and **(C)** Jurkat cells. **(D–E)** Screening of the second site in **(D)** CHO-K1 and **(E)** Jurkat cells. **(F)** Screening of the third site in CHO-K1 cells. **(G, H)** Validation of selected mutants in **(G)** Jurkat cells and **(H)** PBMCs. **(I, J)** Validation of selected mutants using Fluc reporter gene in **(I) **CHO-K1 and **(J)** PBMCs. n = 3, Mean ± SD; *p value < 0.05, **p value < 0.01, ***p value < 0.001, ****p value < 0.0001, ns represents no significance.

The wild-type (WT) *JL* transposase specifically recognizes the ITR, composed of 28 bp bases. Mutations in the ITR may affect transposase recognition, thereby influencing transposition and integration efficiency (**Figure 3A**). To evaluate the impact of mutations in the ITR region of the *JL* transposon on transposition efficiency, we constructed 6 plasmids containing EGFP as a reporter gene, in which the first CpG site of the *JL* transposon ITR was modified to eliminate the CpG dinucleotide. The expression of the target gene, EGFP, was assessed in CHO-K1 and Jurkat cell lines, with the EGFP positive rates measured *via* flow cytometry at 12 days post-transfection.

The results showed that in the CHO-K1 and Jurkat cell line, the mutation of the first CpG site to ApG yielded the highest EGFP positive rate (**Figures 3B, C**), significantly surpassing that of the premutation group. Thus, mutating the first CpG site of the *JL* transposon ITR to ApG markedly enhanced target gene expression, providing a foundation for further mutations based on this modification.

Subsequent screening focused on plasmids with the second site mutated to TpG (**Figures 3D, E**), followed by a third round of mutation screening. The group with the third site mutated to CpT had the highest EGFP positive rate among all experimental groups (**Figure 3F**). Although the positive rate of the CpT group remained lower than that of the WT, it exhibits a certain degree of integration capability. Therefore, the third site was temporarily assigned as CpT, with future tests planned to evaluate the stability of other reporter genes. We also attempted other mutations, but they did not yield favorable results (**Supplementary Figure S2**). This suggests that the third site may be critical for transposase recognition. In summary, through iterative mutation screening, low-CpG optimized variants of the *JL* transposon ITR region were identified, with the three CpG sites mutated to ApG, TpG, and CpT, respectively.

### Further validation of the selected *JL* ITR low-CpG mutants’ efficacy

To confirm whether the ITR mutations were effectively recognized by transposase, we evaluated the selected mutants in Jurkat and human peripheral blood mononuclear cells (hPBMCs) (**Figures 3G, H**). All three site mutants stably expressed EGFP in hPBMCs. The positive rates of the three site mutants were significantly higher than those of the WT group.

Next, we incorporated the firefly luciferase (Fluc) reporter gene into the optimized mutants and validated its expression in CHO-K1 cells and hPBMCs using normalized analysis. In CHO-K1 cells, there was no significant difference in Fluc expression between the first site the second mutant and the WT group (**Figure 3I**). In hPBMCs, Fluc expression from the first and second site mutants was significantly higher than that of the WT group, whereas the third site mutant showed reduced expression relative to the WT (**Figure 3J**). Additionally, the Fluc expression in mutated plasmids was superior in hPBMCs compared to CHO-K1 cells. This demonstrated that the mutations at the first two sites significantly enhanced the integration ability of the target gene in different cell lines, while the mutation at the third site might negatively impact integration efficiency.

### LCG CAR-T cells demonstrated enhanced anti-tumor efficacy *in vitro*

**Figure 4:**
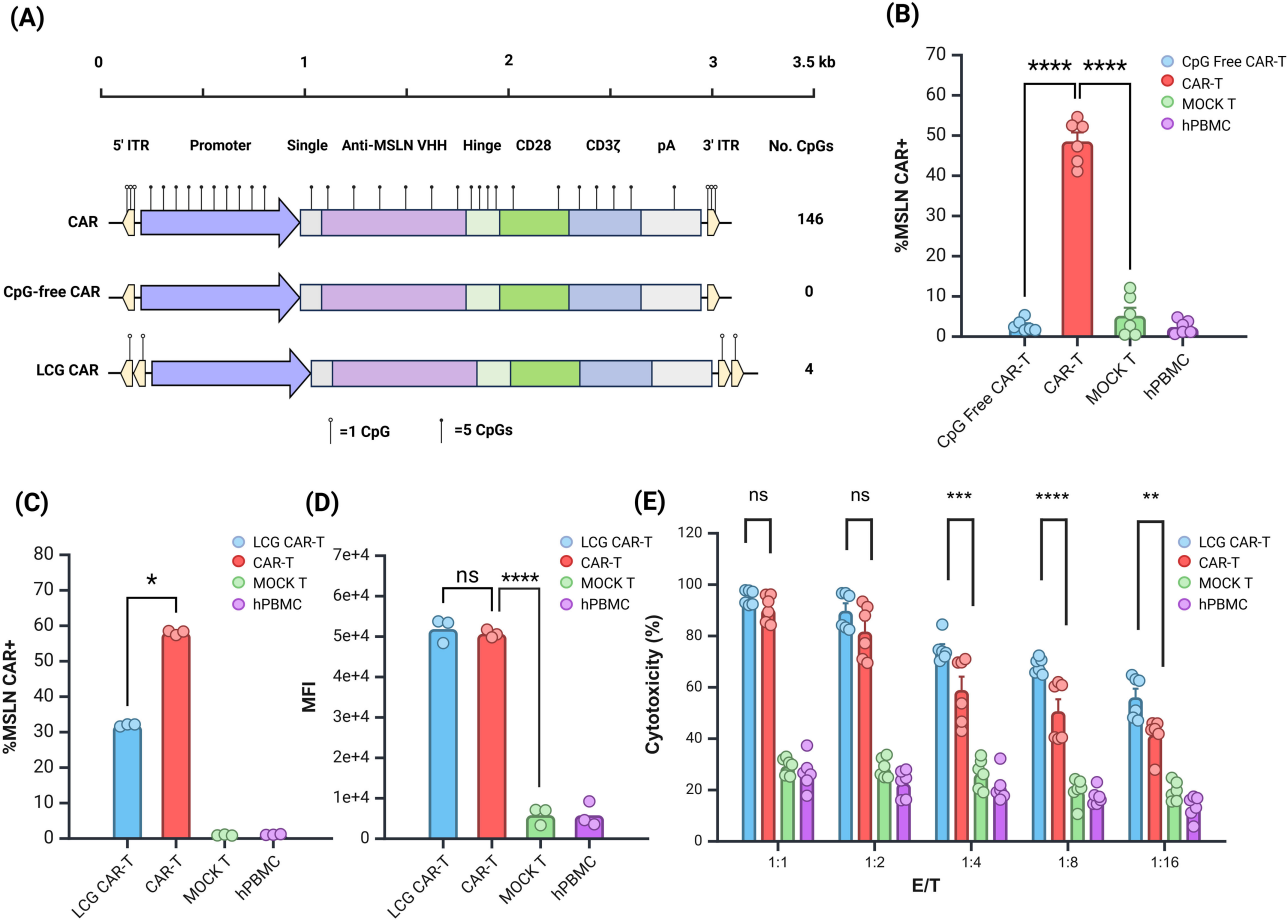
Evaluation of Anti-tumor Effects of LCG CAR-T Cells *In Vitro*. **(A)** Schematic of CAR structure. **(B)** CpG-free CAR positivity rate. **(C, D)** LCG CAR positivity rate **(C)** and MFI **(D)**. n = 3. **(E)** Cytotoxic of LCG CAR-T cells on SK-OV-3-Luc tumor cells *in vitro*, n = 6, mean ± SD; *p value < 0.05, **p value < 0.01, ***p value < 0.001, ****p value < 0.0001, ns represents no significance.

In line with transgene optimization strategy, we eliminated CpG sites from the MSLN CAR gene and promoter, and performed codon optimization to remove codons that might negatively impact transgene expression. The plasmid structure is illustrated in **Figure 4A**.

Initially, we evaluated the *JL* transposon system lacking all three CpG sites in hPBMCs. CpG-free CAR-T cells resulted in a significantly lower positive rate compared to normal CAR-T cells (**Figure 4B**). This suggests that stable integration was compromised, consistent with earlier findings using the EGFP and Fluc reporter genes. The third CpG site appears critical for transposase recognition and cannot be mutated further at this time. Future CAR-T cell preparations will leverage plasmid vectors modified at the first two CpG sites.

Building on previous experimental insights, the double ITR plasmids exhibited superior transposition efficiency (32). Consequently, we constructed a double ITR plasmid and developed a LCG CAR plasmid based on vectors with mutations at the first and second sites. The CAR-T cells derived from this plasmid were termed LCG CAR-T cells. Although the positive rate of the LCG CAR-T cell group was significantly lower than CAR-T cells (**Figure 4C**), stable integration and expression were achieved, with no significant difference in MFI (**Figure 4D**).

Further comparison of the subgroup differences between LCG CAR-T cells and CAR-T cells revealed no significant differences in the subgroups of CD4+ and CD8+ T cells (**Supplementary Figure S3A**). Notably, LCG CAR-T cells exhibited a higher proportion of CD4+ CAR-T cells, which may enhance IFN-γ secretion.

Using SK-OV-3-Luc cells as the target for MSLN CAR-T cell cytotoxicity, flow cytometry revealed a 97.7% positive rate for MSLN antigen on the surface of SK-OV-3-Luc cells (**Supplementary Figure S3B**). Co-culturing SK-OV-3-Luc cells with CAR-T cells at effector-to-target (E:T) ratios of 1:1, 1:2, 1:4, 1:8, and 1:16 for 24 hours, with hPBMCs serving as controls, allowed for the evaluation of CAR-T cell cytotoxicity against tumor cells. At E:T ratios of 1:1 and 1:2, there was no significant difference in cytotoxicity between LCG CAR-T cells and CAR-T cells at E:T ratios of 1:1 and 1:2 (**Figure 4E**). However, as the E:T ratio decreased, the cytotoxicity of the CAR-T cells declined significantly. At E:T ratios of 1:4, 1:8, and 1:16, the cytotoxicity of LCG CAR-T cells was significantly superior to CAR-T cells (P<0.001). These results suggest that LCG CAR-T cells can specifically recognize and exert anti-tumor effects on cells with high MSLN expression *in vitro*, exhibiting a stronger inhibitory effect on tumor cell growth.

### *In vivo* anti-tumor efficacy of LCG CAR-T cells in a subcutaneous ovarian cancer xenograft model

**Figure 5:**
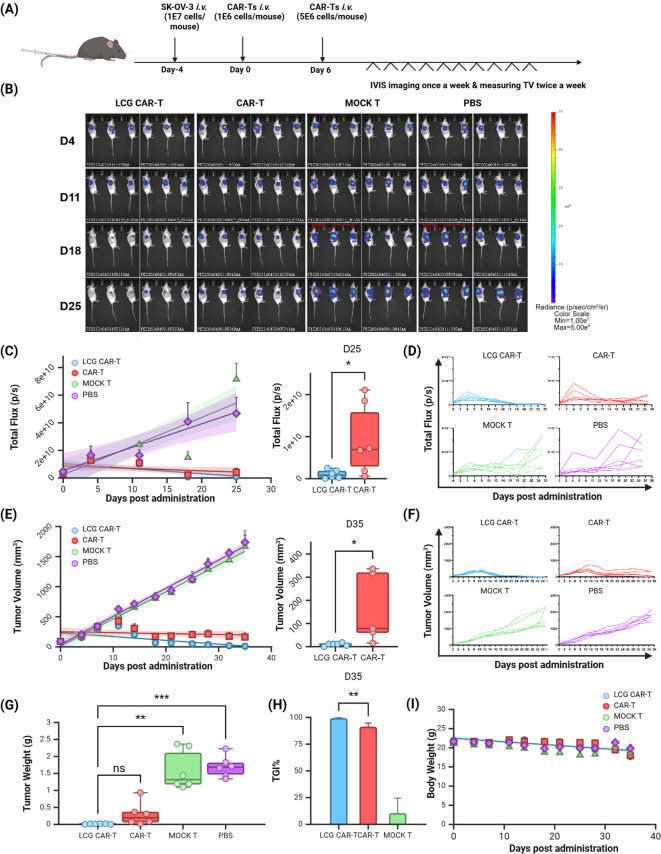
*In Vivo* Anti-tumor Effects of LCG CAR-T Cells in a Human Ovarian Cancer Subcutaneous Xenograft Model. **(A)** Schematic of immunotherapy protocol. **(B)** Bioluminescence imaging at 4, 11, 18 and 25 days post CAR-T cell treatment. **(C, D)** Calculation of tumor total flux or individual data (p/s) using *in vivo* imaging software. **(E)** Tumor volume and **(F)** individual tumor volume (mm3). **(G)** Tumor weight (g). **(H)** Tumor inhibitory rate. **(I)** Body weight changes. n = 6, mean ± SEM; *p value < 0.05, **p value < 0.01, ***p value < 0.001, ns represents no significance.

To evaluate the therapeutic efficacy of LCG CAR-T cells against solid tumors, we established an NSG mouse model bearing SK-OV-3 ovarian cancer tumors, with the immunotherapy regimen illustrated in **Figure 5A**.

*In vivo* imaging demonstrated a continuous increase in tumor fluorescence intensity in both the MOCK T cell and PBS groups (**Figure 5B**). Quantitative analysis revealed that the total fluorescence intensity in the MOCK T cell and PBS groups was significantly higher compared to the experimental group and the positive control group treated with LCG CAR-T cells. Notably, on Day 25, the fluorescence intensity in the experimental group was significantly lower than CAR-T cells (**Figures 5C, D**).

Tumor volume measurements confirmed that tumors in the MOCK T cell and PBS groups continued to grow without significant inhibitory effects (**Figures 5E, F**). Both the LCG CAR-T cells and the CAR-T cells demonstrated significant tumor-suppressive effects. By day 18, tumor volume in the experimental group was already below the baseline measured on day 0, indicating a more pronounced tumor regression effect mediated by LCG CAR-T cells. By day 35, tumor volume in the LCG CAR-T cells was significantly lower than in the conventional CAR-T cells (without ITR mutation) group, underscoring the enhanced tumor-suppressive efficacy of LCG CAR-T cells.

Upon conclusion of the experiment on Day 35, tumor samples were collected and weighed. The LCG CAR-T cells exhibited the lowest tumor weight, significantly lower than that of the MOCK T and PBS groups (**Figure 5G**) and LCG CAR-T cells mediated a more significant tumor inhibitory rate (**Figure 5H**). Monitoring body weight changes indicated that mice treated with LCG CAR-T cells and CAR-T cells maintained stable body weight (**Figure 5I**). There was no significant change in organ weight in mice (**Supplementary Figures S4A-E**). These findings are consistent with the results of total fluorescence intensity and tumor volume measurements, indicating that LCG CAR-T cells demonstrate stable and enduring tumor-suppressive effects.

### Modified CAR-T cells enhance T cell infiltration in a subcutaneous ovarian cancer model

**Figure 6:**
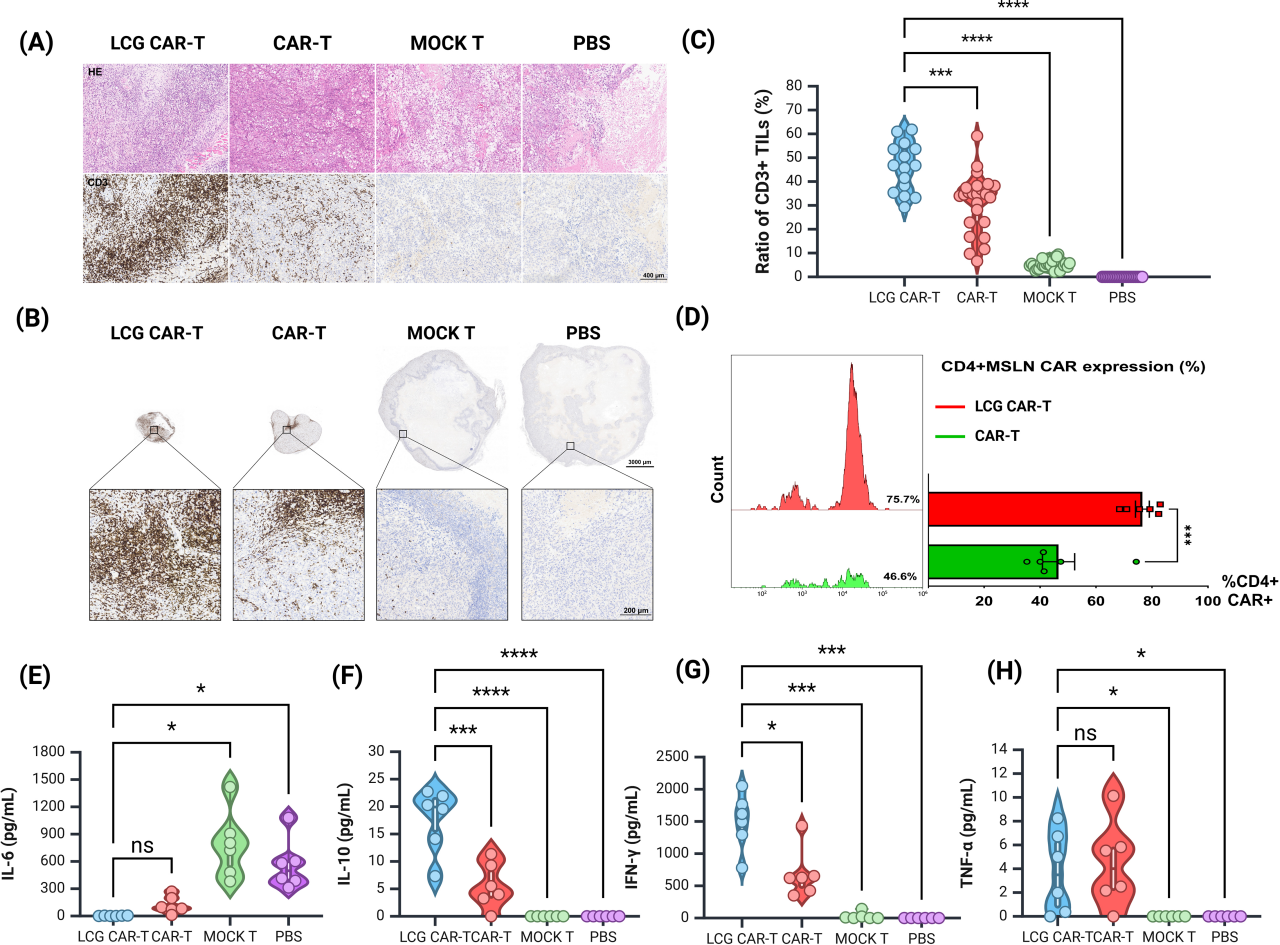
Histological and cytokine assessment of tumor in a human ovarian cancer subcutaneous tumor model. **(A)** H&E staining and CD3 immunohistochemistry of tumor sections. Scale bar = 400 μm. **(B)** Measurement of tumor-infiltrating T cells (CD3+) in tumor sections by IHC. Top: Representative IHC images, scale bar = 1000 μm. Bottom: Magnified view, scale bar = 200 μm. **(C)** Quantification of CD3+ TILs in tumors by IHC. **(D)** Proportion of CD4+ CAR-T cells in mouse peripheral blood by FACS. **(E, H)** CBA detection of **(E)** IL-6, **(F)** IL-10, **(G)** IFN-γ, and **(H)** TNF-α levels in serum of different groups of mice. n = 6, mean ± SEM; *p value < 0.05, ***p value < 0.001, ****p value < 0.0001, ns represents no significance.

To evaluate the enhanced anti-tumor efficacy of LCG CAR-T cells, we quantified the proportion of tumor-infiltrating lymphocytes (TILs) positive areas. IHC was utilized to assess CD3 expression in tumors treated with LCG CAR-T cells, conventional CAR-T cells, and MOCK T cells (**Figures 6A, B**).

The LCG CAR-T cell group demonstrated a significantly higher T cell aggregation (46.43 ± 2.58%) compared to the CAR-T cells (31.11 ± 2.52%) and the MOCK T cells (5.28 ± 0.39%) (**Figure 6C**). The positive area ratio of TILs in the LCG CAR-T cell group was markedly elevated relative to the conventional CAR-T cells and MOCK T groups (**Figure 6C**), indicating a pronounced accumulation of T cells surrounding the tumor cells. These results further substantiate the superior therapeutic efficacy of LCG CAR-T cells.

### The improved anti-tumor efficacy is correlated with the persistence of CD4+ CAR-T cells and the levels of inflammatory cytokines

Peripheral blood samples from tumor-bearing mice were stained with CD3, CD4, and VHH nanobody flow antibodies on D25. The proportion of CD4+ CAR-T cells in the LCG CAR-T group was significantly higher (75.7%) than in the conventional CAR-T group (46.6%) (P = 0.0007), corroborating *in vitro* results (**Figure 6D**). Peripheral blood T cell populations in mice were shown in **Supplementary Figure S4F**.

Subsequently, serum was isolated from the peripheral blood of mice to detect cytokine levels. The expression level of IL-6 in LCG CAR-T cells was lower compared to the conventional CAR-T cells and MOCK T groups, while IL-10 and IFN-γ levels were significantly elevated (**Figures 6E–H**). The marked increase in IFN-γ in LCG CAR-T cells suggests enhanced tumor inhibitory activity, as CD4+ CAR-T cells can induce tumor cell death at distant sites in an IFN-γ-dependent manner (33). In contrast, the hunching behavior observed in the MOCK T cell group may be attributed to low IL-10 expression coupled with elevated IL-6 levels, leading to an inflammatory response. Overall, our findings indicate that LCG CAR-T cells reduce inflammation-related cytokine levels in mouse plasma compared to conventional CAR-T cells.

## Discussion

The recognition and binding ability of transposase to ITRs affects transposition efficiency. Barbara Scheuermann et al. attempted to introduce point mutations in the ITR of the SB transposon system. They hypothesized that the common ITR sequences of transposons in the *Salmo salar* genome could increase transposition efficiency. However, their results demonstrated that such point mutations did not yield an increase in transposition efficiency in transfected HeLa cells (34). In this study, the transgene expression ability of the *JL* transposon system significantly decreased after the removal of three CpG sites in the ITR. Removing the first two CpG sites in the ITR increased the expression of EGFP and Fluc reporter genes in CHO-K1 cells, Jurkat cells, and hPBMCs, illustrating that mutations within the ITR can significantly influence transgene expression levels.

Garrison BS et al. studies have indicated that incorporating an additional pair of inverted terminal repeat sequences (double ITR) adjacent to the ITR can markedly enhance the efficiency of transgene integration, thereby achieving sustained and high-level protein production (32). Therefore, our study employed a double ITR strategy in the construction of CAR plasmids. Yun Haeng Lee et al. found that most integrated transposons underwent at least some degree of DNA methylation (35). One possible mechanism is that cells mark transposon sequences rich in CpG dinucleotides as foreign genes, thereby targeting them for silencing. The trend of transgene silencing is consistent with increased CpG methylation of transposons. Additionally, many silenced clones can be reactivated in the presence of the DNA methyltransferase inhibitor 5-azacytidine (Azacitidine, 5-AzaC). Notably, double ITRs appear to respond more favorably to DNA methylation inhibitors, suggesting that the combined application of double ITRs and DNA methylation inhibitors could effectively enhance protein yield in transgenic systems (32).

Our work found that LCG CAR-T cells can mediate an increase in the proportion of CD4+ CAR-T cells and significantly enhance IFN-γ secretion. IFN-γ directly promotes apoptosis in tumor cells, and CD4+ CAR-T cells can facilitate tumor killing at distant sites in an IFN-γ-dependent manner (33). Morgane Boulch et al. found that the production of IFN-γ is the main mechanism by which anti-CD19 CD4+ CAR-T cells clear tumors. Mechanistically, CD4+ CAR-T cells serve as a main source of IFN-γ, which can act on tumor cells from a distance, selectively inducing apoptosis in tumors sensitive to cytokine-driven cell death, including antigen-negative variants. Different tumor models exhibit varying sensitivities to IFN-γ-induced apoptosis (36–38). For instance, in models such as OVCAR3 and B16 melanoma, CD4+ CAR-T cells effectively mediate tumor regression; however, this is not the case in models like MC38. The intrinsic sensitivity of tumor cells to IFN-γ-induced apoptosis is a major determinant of CD4+ CAR-T cell therapy. In our study, both *in vitro* experiments using SK-OV-3-Luc cells and *in vivo* models demonstrated that LCG CAR-T cells possess superior tumor-killing and regression rates compared to conventional CAR-T cells. The levels of IFN-γ and CD4+ CAR-T cells in the peripheral blood of mice were significantly higher than those of conventional CAR-T cells, which may prove that CD4+ CAR-T cells can mediate tumor killing in an IFN-γ-dependent manner, and SK-OV-3 tumor cells may be sensitive to IFN-γ-induced apoptosis. Recent studies have also shown that IFN-γ can increase contact-dependent killing by CAR-T cells through the upregulation of adhesion molecules, such as ICAM-1, on tumor cells (39, 40). Additionally, IFN-γ plays a role in sustaining the cytotoxic function of CAR-T cells (33, 41), which is consistent with the significant increase in TILs observed in this experiment.

In addition, LCG CAR-T cells also mediate significant increase in IL-10 levels and a decrease in IL-6 levels in mice. IL-10 is an anti-inflammatory factor that inhibits the release of IL-6, IL-8, and TNF-α to exert anti-inflammatory effects. It can also promote the proliferation and activation of CD8+ T cells, releasing higher levels of IFN-γ to achieve anti-tumor effects (42). Conversely, IL-6 acts as a pro-inflammatory cytokine that enhances macrophage maturation and activation while regulating various physiological processes, including the acute phase response, thermogenesis, glucose metabolism, and appetite (43, 44). IL-6 blockade can reverse most cytokine release syndrome (CRS) symptoms and downregulate cytokines in many patients (45, 46). Chronic inflammatory responses caused by some pro-inflammatory factors may promote tumor growth. In preclinical models, IL-6 has also been found to be associated with CRS-mediated mortality (47) and promotes macrophage activation by inducing nitric oxide synthase and nitric oxide production (48). In this study, LCG CAR-T cells reduced the inflammatory response *in vivo* by suppressing pro-inflammatory cytokine levels.

Overall, our results indicate that LCG CAR-T cell therapy surpasses conventional CAR-T cell therapy in terms of anti-tumor efficacy, inflammatory reduction, and T cell infiltration. The construction of LCG CAR-T cells represents a promising strategy to enhance CAR-T cell effectiveness. This study’s identification of low-CpG ITR mutants within the *JL* transposon system, combined with CpG-free CAR genes and promoters, has led to the development of CAR-T cells with improved anti-tumor activity, offering new avenues for gene therapy and genetic tool development.

## Data Availability

The original contributions presented in the study are included in the article/**Supplementary Material**. Further inquiries can be directed to the corresponding authors.
