# Supplementary figures and images for: Unleashing the potential of a low CpG Passer transposon for superior CAR-T cell therapy
Source: Front Immunol. 2025 Feb 6;16:1541653. doi: 10.3389/fimmu.2025.1541653 (PMC11840574; doi:10.3389/fimmu.2025.1541653)

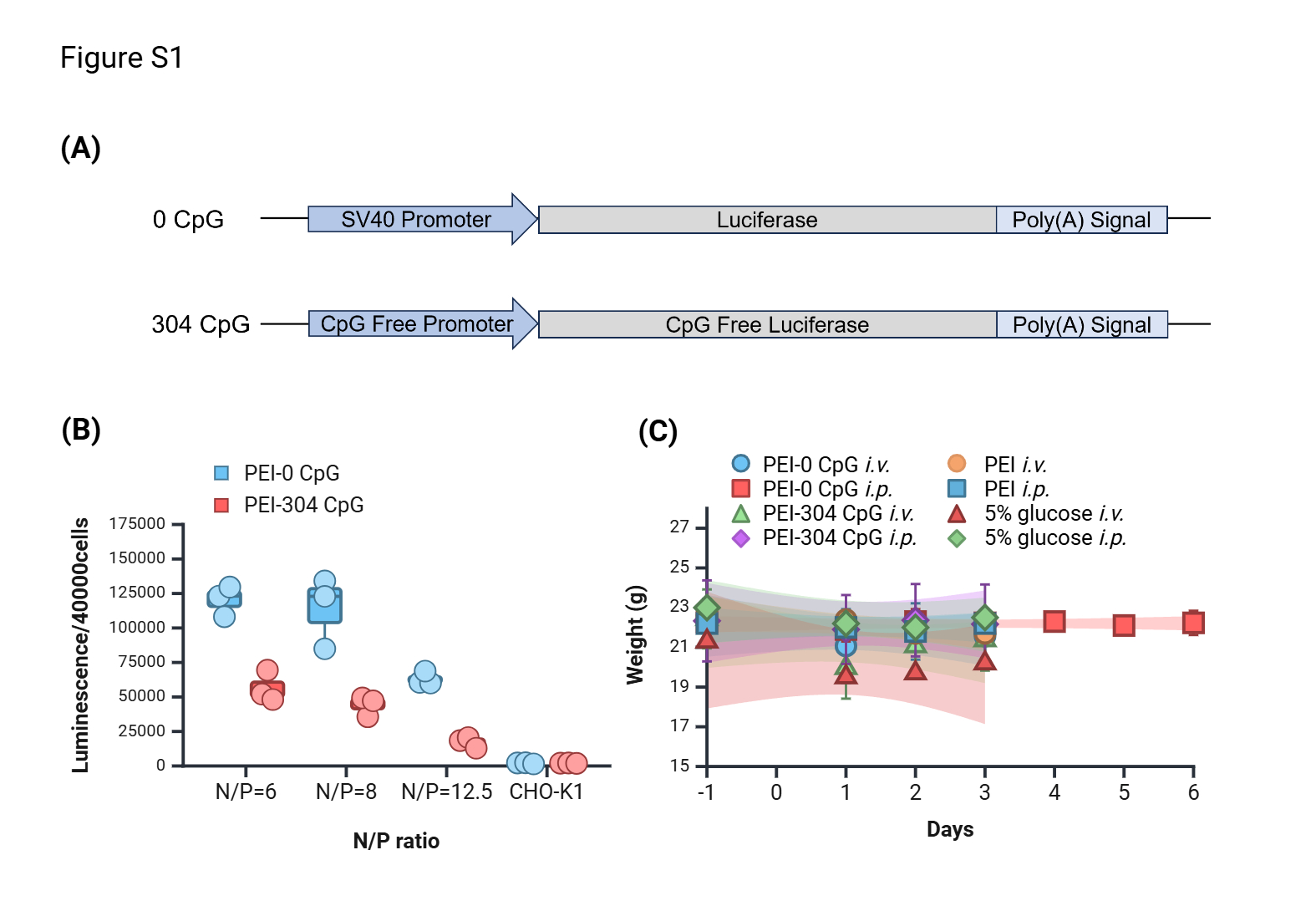

Supplement: Supplementary file 1 [file Image1.jpeg]

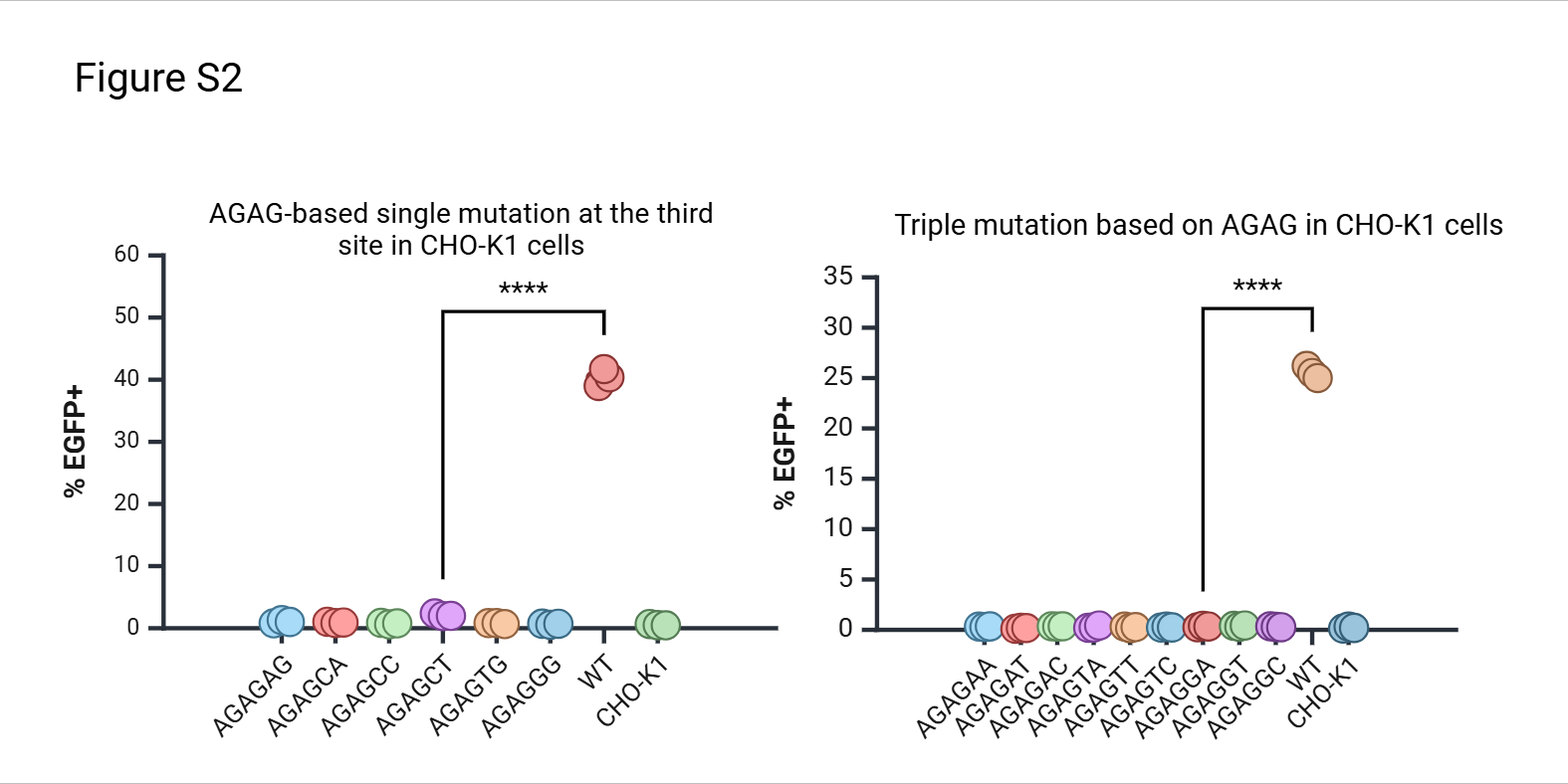

Supplement: Supplementary file 2 [file Image2.jpeg]

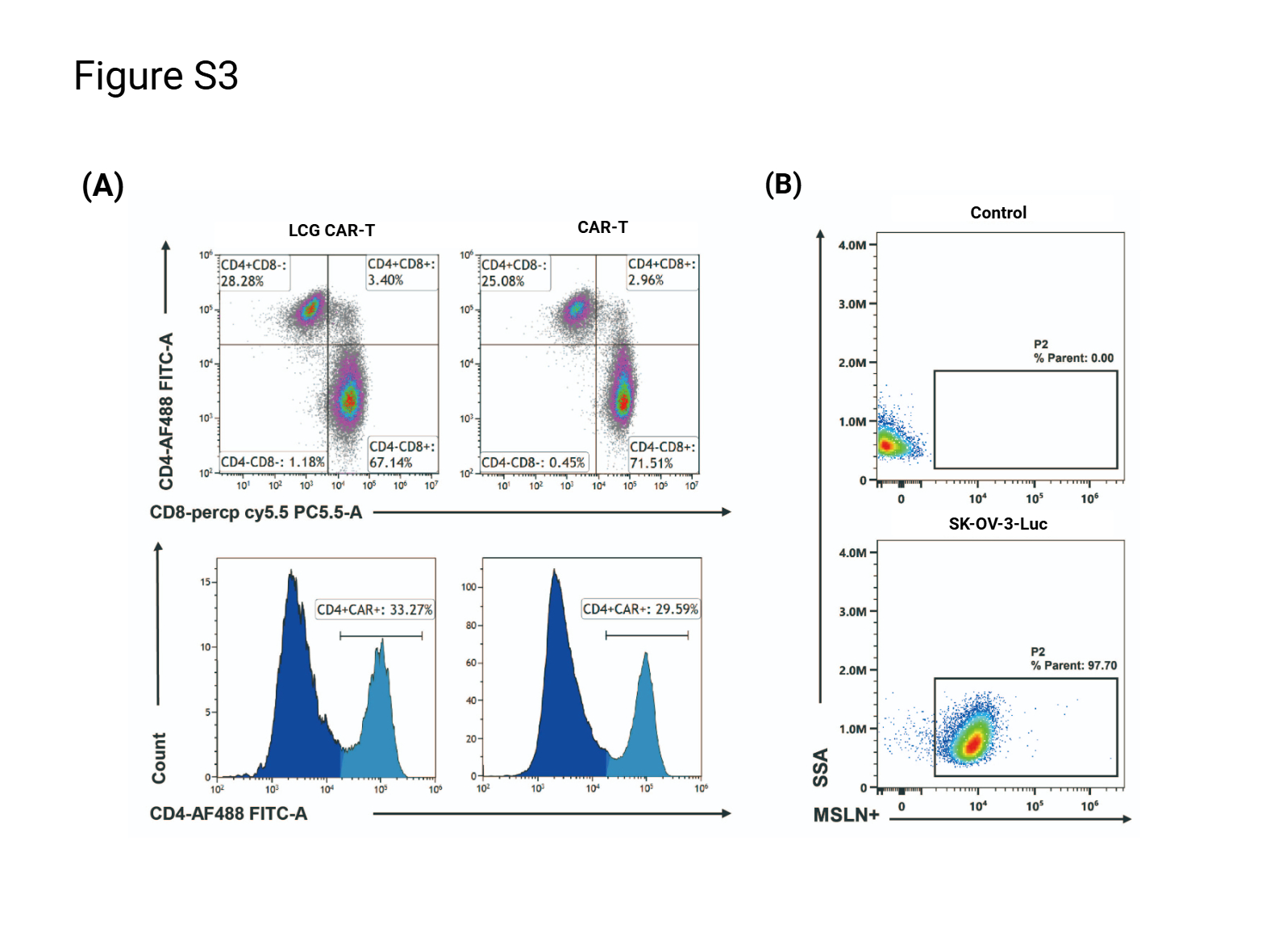

Supplement: Supplementary file 3 [file Image3.jpeg]

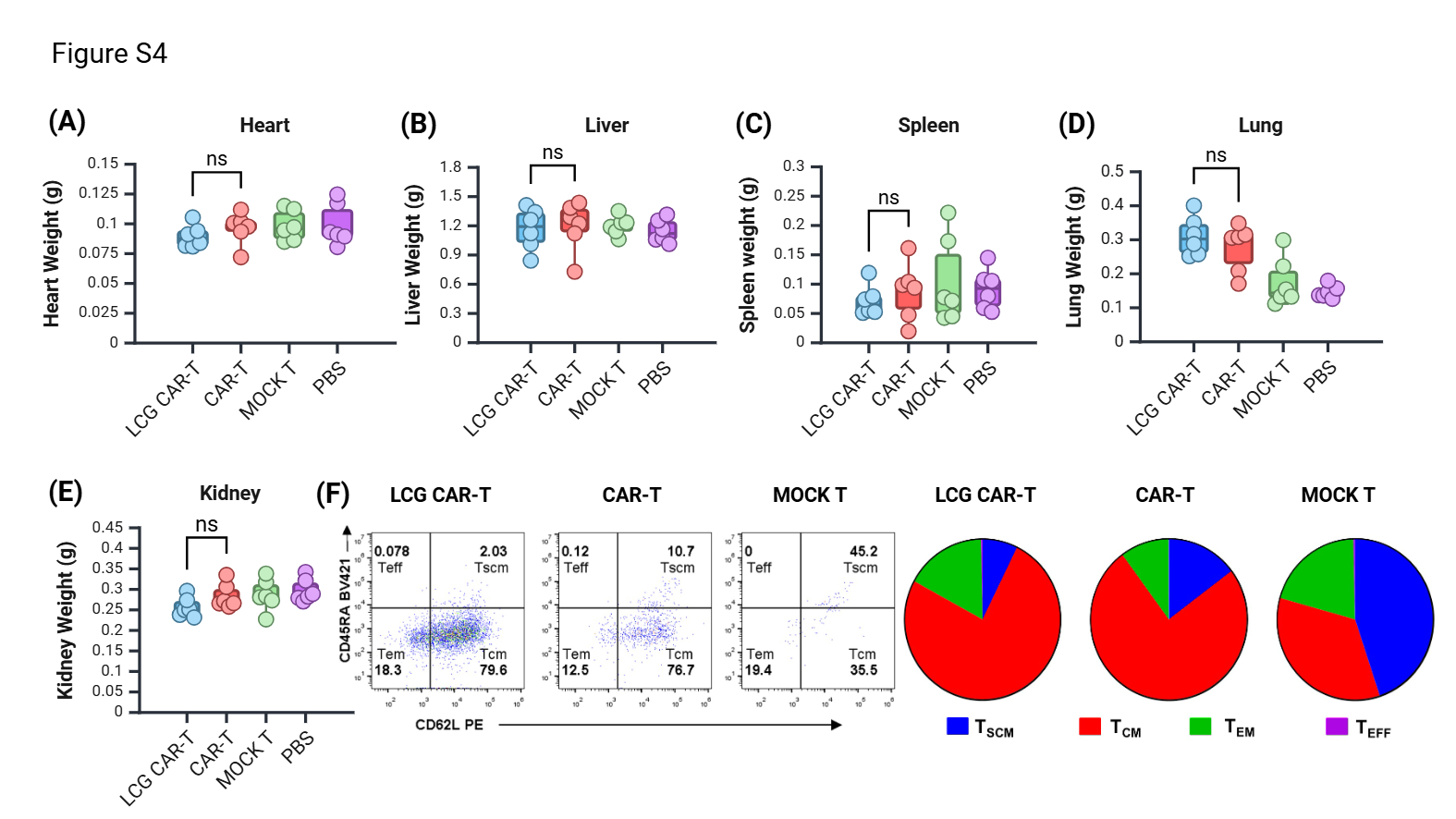

Supplement: Supplementary file 4 [file Image4.jpeg]
